# Can Land Marketization Help Reduce Industrial Pollution?

**DOI:** 10.3390/ijerph16122213

**Published:** 2019-06-22

**Authors:** Weidong Sun, Zhigang Chen, Danyang Wang

**Affiliations:** 1 School of Geography and Ocean Science, Nanjing University, Nanjing 210023, China; sunweidong94@163.com (W.S.); Wangdanyang@smail.nju.edu.cn (D.W.); 2 Key Laboratory of Coastal Zone Exploitation and Protection, Ministry of Natural Resources of China, Nanjing 210017, China

**Keywords:** industrial land, market-oriented reform, land price, industrial pollution, containment effect

## Abstract

Industrial pollution control is a difficult problem in China’s current economic transformation, and the Chinese government has implemented many measures to deal with it. However, little research has focused on the relationship between land policy and industrial pollution. Based on the theoretical discussion of the mechanism influencing the market-oriented reform of industrial land (mainly refer to the marketization of land conveyance price and the openness of land conveyance process) on urban industrial pollution, we constructed an analytical framework by linking land policy with industrial pollution. Then, we constructed an econometric model and chose the statistical data of 104 large- and medium-sized cities in mainland China from 2003 to 2016. The results indicate that with the marketization of the industrial land conveyance price, urban industrial pollution is presenting an inverted U-shaped change trend. For cities in different development stages of industrialization, there is no difference in the impact of industrial land conveyance price on urban industrial pollution. However, the openness of industrial land conveyance promotes and inhibits the urban industrial pollution in the stages of industrialization and post-industrialization, respectively. Finally, this paper puts forward some suggestions on how to control industrial pollution from the perspective of further improving the industrial land conveyance mechanism.

## 1. Introduction

Pollution caused by human economic activities has seriously harmed the global environment [1], and environmental problems are a huge public concern [2]. Since China’s reform and opening up, rapid industrialization and urbanization have driven the continuous and stable growth of the country’s economy [3] and China’s industrial economy is continuing to expand. In 2015, China’s total manufacturing output accounted for 22% of the world’s total, ranking first in the world. However, the rapid development of industry has simultaneously resulted in serious environmental pollution problems. From 2000 to 2015, the total emissions of various industrial pollutants in China continuously increased. The total emissions of industrial waste water and industrial waste gas increased by 530 million tons and 5.50 × 10^4^ billion m^3^, respectively, and the amount of industrial solid waste increased by 2495 million tons [4], seriously threatening residents’ health [5,6] and the sustainable development of the economy [7,8]. The pollution from industrial production is a result of the economic behavior of industrial enterprises to a large extent [9], and changes in its scale and degree are not only directly affected by the government’s administrative control measures [10], but also by relevant economic incentive policies [11,12]. Market mechanisms are typically used as economic incentive policies. The introduction of a market mechanism to the allocation of industrial land not only affects the economic behavior of industrial enterprises, but also indirectly affects the pollution emissions and governance behavior of enterprises. Therefore, we attempted to link the marketization of industrial land allocation with industrial pollution to further discuss the relationship between the two. Our findings help to deeply understand the dynamics behind the generation of and changes in industrial pollution, and are important for effectively controlling pollution and improving the urban environmental quality.

Many have studied the factors that influence and the mechanisms that drive urban industrial pollution. The factors affecting the generation of and changes in urban industrial pollution can be classified into two categories: micro and macro factors. Macro factors mainly involve economic development, industrial structure and industrial agglomeration, environmental regulation, and foreign investment. From the perspective of economic development, it is generally thought that there is an inverted U-shaped relationship between environmental pollution and per capita income. That is, when the economy develops to a certain level, the increase in per capita income contributes to the improvement of environmental quality [13,14,15,16]. As for the impact of industrial structure and industrial agglomeration, Zhou et al. [17] and Cheng et al. [18] stated that upgrading and optimizing industrial structure could effectively reduce pollution emissions. However, He et al. [19] reported that the upgrading of industrial structure brought about by the introduction of enterprises may lead to the deterioration of the urban environment. Studies have shown that industrial agglomeration will generally lead to an increase in total pollution emissions [20,21], but will also reduce the intensity of pollution emissions due to the scale economy [22,23]. Li [24] and Yang [25] found that a turning point exists between industrial agglomeration and environmental pollution, the relationship between which is not a simple linear relationship. In terms of the impact of environmental regulation and foreign investment, strict environmental regulation could force high-polluting enterprises to withdraw [26,27], but may also prompt product and production process innovation [28] to reduce environmental pollution. Sapkota and Bastola [29] discovered that the introduction of foreign capital adversely affects the local environmental quality, but other studies have found that it also improves the environmental quality of recipient countries or regions, as a result of the cleaner energy use and more efficient energy use of foreign-funded enterprises [30,31]. Due to China’s special economic system, local governments’ behavior affects urban industrial pollution emissions [32,33]. Under the pressure of economic competition and financial incentives, local governments tend to attract industrial enterprises through tax incentives and other measures, which often exacerbates local industrial pollution [34,35]. Micro factors are mainly related to specific industrial enterprises and their characteristics, such as their size, production efficiency, the application of new production processes, and production technology [36,37]. Cole et al. [38,39,40] discovered from their case study in the United Kingdom, China, and Japan that enterprise size, energy use, capital and labor intensity, research and development expenditure, quantity of export commodities, and public awareness are the main factors affecting industrial pollution emissions. Some studies have focused on the differences in the pollution emissions of industrial enterprises with different forms of ownership. The pollution emission intensity of foreign enterprises and private enterprises is lower than that of state-owned enterprises, and enterprises that are larger, with more exports, and a more educated labor force usually have lower pollution emissions [41]. As can be seen from the above studies, the existing research focused less on the relationship between the allocation of urban land and industrial pollution. Both macro factors (e.g., industrial characteristics, institutional policy) and micro factors (e.g., enterprises’ characteristics, production behavior) are closely related to the allocation of industrial land. Therefore, when exploring the factors influencing industrial pollution, the impact of land supply and allocation (especially the marketization of industrial land allocation) cannot be ignored.

In this study, we discussed the specific impact of industrial land marketization on industrial pollution. The remainder of this paper is organized as follows. In Section 2, we briefly introduce the market-oriented reform process of China’s industrial land, and then theoretically discuss the impact. In Section 3, we construct an econometric model based on theoretical analysis, and then conduct a brief descriptive statistical analysis. In Section 4, taking 104 Chinese cities as the research sample, we evaluate the specific impact based on the above econometric model, and then explain and analyze the results. In the last section, we provide both conclusions and related policy recommendations.

## 2. Theoretical Framework

### 2.1. China’s Industrial Land Market Development

Following the reform and opening up of China in 1978, with the promotion of the reform of the socialist market economy system, the institutional arrangement of urban land conveyance (churang), which is “free, indefinite, and non-flowing” under the traditional planned economy system, has been gradually replaced by a “compensated, limited, and mobile” system [42], and the urban land market has been formed and developed. Although the reform of the urban land use system began with the demand for industrial land by foreign-funded enterprises, China’s urban land market was not commonly acknowledged until Shenzhen City first transferred the use rights of state-owned land for residential use by means of auction in December 1987. Since then, the development of the urban land market has mainly focused on the compensated conveyance of profit-oriented land, whereas the market-oriented reform of industrial land has lagged behind the development of the urban land market.

**Figure 1:**
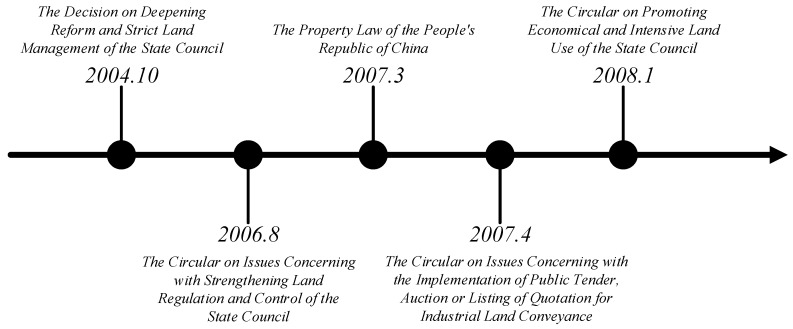
Policy evolution of the market-oriented reform of industrial land in China.

Beginning in the 1990s, with the widespread implementation of the land compensation conveyance system in China, the urban land market system preliminarily formed through cultivation and standardization. The tremendous asset effect of land and its role in promoting the development of the national economy have become increasingly apparent. However, the compensated industrial land was mainly conveyed by means of negotiation in this period, and large amounts of rent-seeking behavior and malversation occurred in the process of land conveyance [43]. Therefore, the market for industrial land conveyance was in disorder [44]. In the context of economic competition between cities and counties, to attract investment, local governments have depressed the price of industrial land and even offered industrial land with a zero price or a negative price. As a result, the price of industrial land is generally low nationwide and the waste of industrial land is serious. The above situation shows that the basic role of the market mechanism in the process of industrial land allocation has not yet been fully realized, and the development of the industrial land market has been sluggish. In response to this situation, after entering the 21st century and on the basis of further promoting the market-oriented reform of the profit-oriented land conveyance, the Chinese government has paid increasing attention to the development of the industrial land market (Figure 1). “The Decision on Deepening Reform and Strict Land Management of the State Council” in 2004 proposed for the first time that, “It is necessary to create conditions for the industrial land to be conveyed gradually by means of tender, auction and listing”. “The Circular on Issues Concerning with Strengthening Land Regulation and Control of the State Council” in 2006 further specified that “industrial land must be conveyed through tender, auction, or listing, and the conveyance price shall not be lower than the minimum price standard announced” [45]. The notice firstly included industrial land conveyance within the scope of open auction. In 2007, “The Circular on Issues Concerning with the Implementation of Tender, Auction or Listing for Industrial Land Conveyance” issued by the Ministry of Land and Resources (MLR) and the Ministry of Supervision (MS) further detailed the policies and measures for the tender, auction, and listing of industrial land conveyance. In the same year, the two ministries also conducted law enforcement supervision of the industrial land conveyance nationwide. “The Property Law of the People’s Republic of China” promulgated in 2007 further upgraded the tender, auction, and listing of industrial land conveyance from a national policy to a national law. “The Circular on Promoting Economical and Intensive Land Use of the State Council” in 2008 demanded that the compensatory conveyance system be completely reformed, and that industrial and commercial lands must be conveyed through tender, auction, or listing. This series of relevant regulations on the conveyance of industrial land has strengthened the fundamental role of the market mechanism in the process of industrial land conveyance. For the conveyance of industrial land, the industrial land price and user must be determined through tender, auction, or listing. Judging from the practical experience of the market-oriented reform of industrial land across China, this reform has played an important role in effectively controlling the total supply of industrial land, curbing the low-price competition of industrial land and improving the land use efficiency [46].

From the evolution process of industrial land marketization reform mentioned above, the demand for industrial land marketization stems from the expansion of industrial land. However, due to the imperfection of the system and the fierce economic competition between local governments, industrial land conveyance has long been based on negotiation. The market mechanism has not yet played a fundamental role in industrial land allocation. The marketization reform of industrial land has lagged behind urban land market development for a long time, and this has led to chaos in the industrial land market. The loss of state-owned land assets and the inefficiency of land use are becoming increasingly serious. To solve these problems, the market-oriented reform of industrial land has been promoted. From 2006 to 2007, the proportion of the area of industrial land conveyed through tender, auction, or listing increased from 3.25% to 26.02%, reaching 82.69% in 2008. From the perspective of the total conveyance scale of industrial land, the average proportion of the conveyance area of industrial land within state-owned land was 53.25% from 2003 to 2016. As such, the impact of the market-oriented reform of industrial land on the development of China’s land market cannot be ignored.

### 2.2. Impact of Industrial Land Marketization on Industrial Pollution

**Figure 2:**
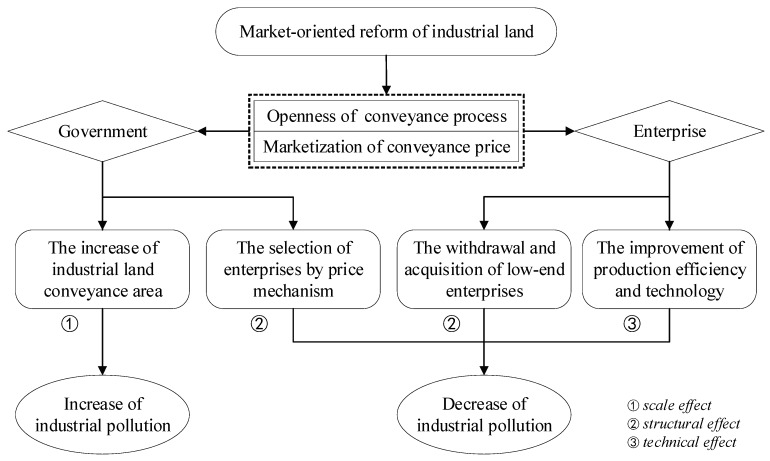
The theoretic framework of urban industrial land marketization and industrial pollution.

The market-oriented reform of industrial land in China can be summarized into two aspects: the openness of the industrial land conveyance process and the marketization of the industrial land conveyance price. The impact of the reform of the decision-making of land allocation and use for the supply (government) and demand (enterprise) of industrial land will affect industrial pollution to varying degrees (Figure 2).

Firstly, from the perspective of the government, since China’s urban land supply is largely controlled by the government, its land supply behavior affects urban industrial pollution emissions by affecting urban economic development and industrial structure. The marketization of industrial land will produce an increase in industrial land, which will stimulate the industrial land conveyance behavior of local governments to a certain extent, with land conveyance fees as the main fiscal revenue. As a rational economic body, the local government may increase the industrial land conveyance area to maximize the benefits (to obtain more land conveyance profits), so that urban industrial pollution will increase with expanding industrial scale. Simultaneously in the early stages, to rapidly develop the economy, the expansion of the industrial scale has been mainly dominated by heavy industries with higher taxes, which will further worsen the urban environment. In addition, the market-oriented reform of industrial land will increase the openness and transparency of the government’s industrial land conveyance process, which will effectively curb the covert operations between some industrial enterprises and the government in the land conveyance process. Local governments will be restricted from offering industrial land at low or even zero prices in the competition for attracting enterprises’ investment. Thus, the competitive pricing strategy of industrial land conveyance will play a role in screening industrial enterprises in the industrial land market. Since the more competitive enterprises tend to be those with higher production efficiency, better technology, and less pollution, the quality of the urban environment will be correspondingly improved with the introduction of these enterprises.

Secondly, from the perspective of enterprises, the role of the market mechanism in the industrial land conveyance process is gradually strengthened as industrial land is mainly conveyed through tender, auction, or listing rather than negotiation or administrative allocation. The openness of the industrial land conveyance process and the marketization of the industrial land conveyance price will affect urban industrial pollution from two aspects. On the one hand, with the rising price of industrial land, the pressure of rising land costs will prompt existing enterprises to optimize the input of production factors by improving their production efficiency and production technology, to reduce the impact of the rising land costs, and improve the competitiveness of enterprises. This can effectively reduce urban industrial pollution. On the other hand, the rise in industrial land prices will increase capital input in the early stages of new industrial enterprises. This will promote the rational allocation of land resources among different industrial sectors, and the industrial structure of the city will adjust accordingly. Thus, the emissions of urban industrial pollution will be affected. Specifically, due to the endogenous mechanism for selecting the superior and eliminating the inferior, the increase in the industrial land price will prompt the transfer of scarce land resources to enterprises with higher production efficiency, better technology, and higher added value. Less productive industrial enterprises will be gradually replaced, so the overall industrial structure of the city will be constantly optimized and upgraded, which could effectively reduce urban industrial pollution.

Generally, the market-oriented reform of industrial land adjusts the supply and demand relationship of urban land through the openness of the industrial land conveyance process and the marketization of the industrial land conveyance price, causing a change in the urban industrial structure, which in turn will affect urban industrial pollution in the following ways. First, the local government expands the industrial scale to increase the land conveyance income, thus exacerbating urban pollution (namely scale effect). Second, the price mechanism and the openness of the conveyance process can screen industrial enterprises and affect the entry and exit of local industrial enterprises, thereby adjusting the urban industrial structure and promoting the improvement of the urban environment (namely structural effect). Third, the rising production costs guide industrial enterprises to increase their production efficiency and improve their production technology, thereby reducing pollution emissions (namely technical effect). The above analysis shows that the impact of China’s industrial land market development on urban industrial pollution is still uncertain. Therefore, it was necessary to further examine its specific impact through empirical analysis.

## 3. Model and Data

### 3.1. Model

To examine the specific impact of China’s industrial land market development on urban industrial pollution, based on the above theoretical analysis and combined with the existing relevant research, we built an econometric model from the reality of urban industrial pollution. Before listing the specific model expression, it should be pointed out that the function of mathematical model mainly includes two aspects: interpretation and prediction. The interpretation is mainly to analyze correlation or causation, while the prediction is a kind of estimation or inference of unknown fields based on limited knowledge. In this study, it is mainly to explain the impact of land marketization on urban industrial pollution. In addition, since the specific model form of the impact of institution or policy on industrial pollution is not clear at present, the existing research in related fields usually adopts the similar linear model to detect whether the policy and related factors have an impact on environmental pollution [47,48,49,50,51]. Of course, in the following specific setting process of the model, the specific expression form of the model will be determined by referring to the existing research results.

We regard urban industrial pollution as the explained variable and the market-oriented reform of industrial land as the core explanatory variable, and introduce relevant control variables according to existing research [52,53,54,55,56,57,58,59,60]. The model is set as follows:

(1) $$UIP_{it}=c+\alpha_{j}L_{it}^{j}+\beta_{k}X_{it}^{k}+\mu_{it}$$

where $UIP_{it}$ represents the industrial pollution for city *i* in period *t*. We used the emission intensity (t/10^8^ yuan) of industrial sulfur dioxide (SO_2_) to measure urban industrial pollution, which specifically refers to the emission of industrial SO_2_ per unit of industrial added value. China’s energy consumption is dominated by coal, making it the world’s largest emitter of SO_2_ [61]. As the major air pollutant, SO_2_ has brought acid rain, sulfuric acid fog and other pollution problems, seriously affecting human health and economic development, and has attracted the attention of governments worldwide [62]. SO_2_ is also one of the important pollutant indicators in the emission reduction targets of China’s 13th Five-Year Plan. On the other hand, compared with other pollutants, China’s public statistics provide more detailed and reliable SO_2_ emission data, which can ensure the availability of data and reliability of research results. Therefore, SO_2_ can be used as the representative to characterize urban industrial pollution. At present, there are also many related studies that use SO_2_ to reflect industrial pollution [63,64]. In addition, using the pollution emission intensity to investigate urban industrial pollution can eliminate the impact of urban scale to more accurately reflect the degree of urban industrial pollution. $L_{it}$ includes the indicators reflecting the market-oriented reform of industrial land in period *t* of city *i*; $X_{it}$ represents a group of control variables except the explanatory variable $L_{it}$; c is a constant; $\alpha_{j}$ and $\beta_{k}$ are the estimated coefficients of the variables $L_{it}^{j}$ and $X_{it}^{k}$, respectively; and $\mu_{it}$ is the error term.

The explanatory variable is the market-oriented reform of industrial land (L). This paper mainly reflects the market-oriented reform of urban industrial land through the urban industrial land price (ilp, yuan/$m^{2}$) and the dummy variable (ilm) of the reform of industrial land conveyance mode (full use of tender, auction, and listing). Considering that with the change in urban industrial land price, its influence on urban industrial pollution may also change, the quadratic term of urban industrial land price ($ilp^{2}$) is added to the model. The dummy variables reflecting the reform of urban industrial land conveyance mode were mainly introduced by “The Circular on Issues Concerning with Strengthening Land Regulation and Control of the State Council” from 2006 [65]. Therefore, we assigned the variable ilm in and after 2007 to be 1 and that before 2007 to be 0 to represent the openness and fairness of industrial land conveyance.

As for the control variables (X), according to the existing research on the influencing factors of urban industrial pollution emissions, we selected four main variables to reduce the estimation deviation: economic development, foreign direct investment, energy intensity, and industrial structure. For the economic development variable (pgdp), industrial pollution is always closely related to economic development. In empirical studies for different countries or regions, the relationship between environmental pollution and economic growth may take many different forms [52,53,54,55]: U-shaped, inverted U-shaped, N-shaped, or inverted N-shaped forms. Therefore, when introducing per capita gross domestic product (GDP; unit: yuan), we added a quadratic term and a cubic term of per capita GDP [56] to test whether the Environmental Kuznets Curve was established in China. The impact of foreign direct investment (fdi) on environmental quality may be mainly realized through the scale effect, structural effect, and technical effect. Some studies reported that foreign direct investment has a significant negative impact on China’s ecological environment [57], whereas others think that foreign direct investment is conducive to reducing China’s pollution emissions [58]. Therefore, we also introduced the foreign direct investment (unit: 10^4^ US Dollar) of each city in the control variables to investigate the impact of foreign direct investment on urban industrial pollution emissions. Energy intensity (ene), as a concentrated reflection of the technical level of production, can reflect the energy input and pollution emissions of industrial production [59]. In this study, we selected the electricity consumption of the total industrial output value of 100 million yuan (unit: kW·h/10^4^ yuan) to reflect the energy intensity of each city. The industrial structure (ind) of a region has a direct impact on industrial pollution emissions [60]. Due to the large energy consumption and serious environmental pollution of the industry, the higher the proportion of the industrial output, the higher the industrial pollution emissions. We chose the proportion of the secondary industry output value to gross regional product (GRP) (unit: %) to reflect the industrial structure of the city. To reduce the possible heteroscedasticity in the model and make the data more stable [66], the per capita GDP (pgdp), foreign direct investment (fdi), and energy intensity (ene) are logarithmically processed.

The final refined model is:

(2) $$\begin{array}{cc} \ln poll_{it} & =c+\alpha_{1}\ln ilp_{it}+\alpha_{2}{\left(\ln ilp_{it}\right)}^{2}+\alpha_{3}ilm_{it}+\beta_{1}\ln pgdp_{it}\\ & +\beta_{2}{\left(\ln pgdp_{it}\right)}^{2}+\beta_{3}{\left(\ln pgdp_{it}\right)}^{3}+\beta_{4}\ln fdi_{it}+\beta_{5}\ln ene_{it}\\ & +\beta_{6}ind_{it}+\mu_{it} \end{array}$$

### 3.2. Data Sources and Descriptive Statistics

Considering the representativeness of the study area and the availability of empirical data, we selected 106 large- and medium-sized cities monitored by the Land Price Monitoring Network of China [67] as the research areas. The study period ranged from 2003 to 2016. Due to the partial absence of land price and socio-economic data in Sanya and Lhasa, the final empirical sample included the panel data of 104 large- and medium-sized cities from 2003 to 2016. The required data sources were as follows. The urban industrial pollution and related socio-economic control variables data were derived from the China City Statistical Yearbook 2004–2017 [68]. The data of urban industrial land price were derived from the Land Price Monitoring Network of China. Table 1 provides the descriptive statistics of the major variables in the model.

**Figure 3:**
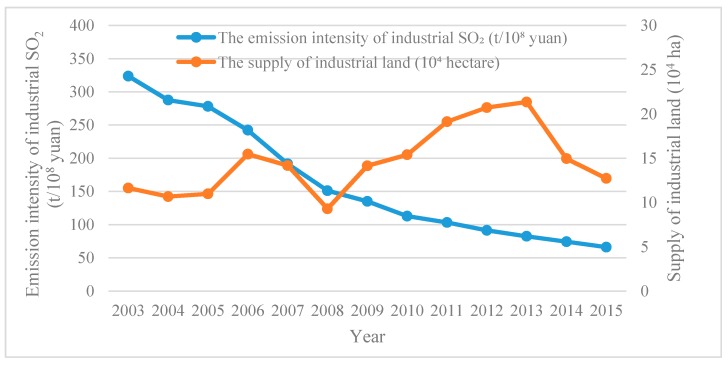
The change in China’s industrial land supply area and industrial pollution from 2003 to 2015.

Before further model estimation, it was necessary to judge the relationship between China’s industrial pollution and industrial land conveyance. As shown in Figure 3, the average emission of industrial SO_2_ showed a downward trend of fluctuations during the study period. The average emission intensity showed a significant downward trend, decreasing from 323.60 t/10^8^ yuan in 2003 to 66.19 t/10^8^ yuan in 2015. This indicates that China’s urban environment has gradually improved in recent years. Further analysis of the changes in industrial land conveyance showed that the supply of industrial land has dropped significantly since 2006, and mainly due to the market-oriented reform of industrial land effectively restricting the government’s land supply, which was conveyed through agreement. Note, due to the lack of industrial land conveyance data from 2009 to 2012, and the proportion of industrial land conveyance area in the total supply area of industrial land always being above 80% during the study period [69,70], we used industrial land supply area to replace industrial land conveyance area. However, the area of industrial land conveyed through tender, auction, and listing increased significantly from 2006 to 2008. After 2008, the supply of industrial land across the country continued to expand, and only more recently did the supply area begin to decrease. After the land marketization reform in 2006, industrial land was conveyed mainly through tender, auction, and listing gradually. At the beginning, with the shrinking supply scale of industrial land, the intensity of industrial pollution emissions continued to decrease. However, when the industrial land supply later increased, the intensity of industrial pollution emissions still continued to decline. This occurred due to the adjustment of production technology of industrial enterprises, but was also related to China’s industrial structure restructuring and the government’s land supply behavior, which was also the main issue that we wanted to explore.

Furthermore, we compared the change in the industrial pollution in the sample cities before and after the market-oriented reform of industrial land in 2006. In 2006 and prior, the average annual industrial SO_2_ emission intensity of the 104 large- and medium-sized cities was 129.23 t/10^8^ yuan, whereas the average annual emission intensity in 2007 and afterward was only 34.09 t/10^8^ yuan. Except for Haikou, the industrial SO_2_ emission intensity of each city significantly reduced. From the perspective of changes in industrial land price, the average annual industrial land price of the 104 large- and medium-sized cities showed an upward trend in the study period, with the average price of each city rising from 353.82 yuan/m^2^ to 609.27 yuan/m^2^. In particular, after the market-oriented reform of industrial land, the industrial land price significantly increased, which is also consistent with the changing trend in urban industrial pollution to a certain extent.

## 4. Results

### 4.1. Estimated Results of Full Sample Model

In this paper, we utilized STATA 13.0 software (StataCorp, Texas, USA) to estimate the sample data of the 104 cities from 2003 to 2016. As the panel data used in this study were different in the estimation of the pooled regression model, fixed effect model, and random effect model, the specific form of the regression model first needed to be determined through testing. The results of the F test and Hausman test showed that the fixed effect model is more effective for the model. The estimated results of the full-sample model are shown in Table 2. Generally, the *R*^2^ and F values of the model were significant, and the model estimation results were good. The specific estimation results of the fixed effect model are mainly analyzed below.

As seen from the estimated coefficients of the control variables, the coefficient of per capita GDP was significantly negative, whereas the coefficient of the second term was significantly positive, and the coefficient of the third term was significantly negative. This indicates that the influence of economic development level (pgdp) on urban industrial pollution (poll) presented an inverted N-shaped change rule. The regression coefficient of foreign direct investment (fdi) for urban industrial pollution was significantly negative, which indicates that foreign direct investment reduced urban industrial pollution to a certain extent, and the hypothesis of a “pollution paradise” [71,72] was not confirmed in China. The influence of the coefficient of energy intensity (ene) on urban industrial pollution was significantly positive, indicating that increases in energy intensity aggravated urban industrial pollution. The industrial structure (ind), represented by the proportion of the output value of the secondary industry, also had a significant positive impact on urban industrial pollution. Urban industrial pollution mainly resulted from the emission of industrial production in the secondary industry. The larger the proportion of the output value of the secondary industry, the greater the impact on urban industrial pollution.

As seen from the estimated coefficients of the main explanatory variables reflecting the market-oriented reform of industrial land in the model, the coefficient of industrial land price (ilp) was significantly positive, and the quadratic term coefficient was significantly negative, indicating that the influence of industrial land price on industrial pollution presented a significant inverted U-shaped change rule. That is, with the increase in industrial land price, urban industrial pollution increased significantly, but when the price of industrial land reached a certain level, the increase in industrial land price significantly reduced urban industrial pollution. Through further calculation, we found that the inflection point of the above inverted U-shaped change rule appears in the industrial land price of about 274 yuan/m^2^ (2.74 million yuan/ha). In other words, when the industrial land price is lower than 274 yuan/m^2^, the increase in industrial land price will encourage local governments to increase the supply scale of industrial land [73], which promotes the expansion of the industrial production scale, and the industries introduced in this stage are mostly pollution-intensive or low-end industries, thus aggravating urban industrial pollution. However, as the price of industrial land continues to rise above 274 yuan/m^2^, the demand for industrial land will be limited by the price mechanism, resulting in a reduction in urban industrial pollution. The increase in industrial land prices will lead to a change in the industrial structure. A higher industrial land price means higher production costs for enterprises, thus facilitating the transformation of industrial production composition from less productive to more productive sectors. In China, this transformation has mainly occurred in the light industry sector with low industrial pollution emissions to the heavy industry sector with high industrial pollution emissions, and then to the high-tech industry with a higher productivity. This change will make urban industrial pollution increase first, and then decrease. This is consistent with the previous theoretical analysis.

The dummy variable ilm, reflecting the market-oriented reform of industrial land conveyance, had a negative influence on urban industrial pollution, but it was not statistically insignificant. This shows that the openness and transparency of industrial land conveyance can reduce urban industrial pollution to a certain extent. However, as the current market-oriented reform process is relatively backward and the local government still has a relatively strong intention to develop heavy industry in this stage of industrialization, the expected emission reduction effect of industrial land market-oriented reform is not completely clear.

### 4.2. Estimated Results of Grouped Sample Model

Based on the estimation of the full sample model and traditional industrial structure theory, we divided the 104 sample cities into two groups according to the industrial structure of each city in 2016. Note, the traditional industrial structure theory mainly judges the stage of industrialization development through the change in the proportion of each industry in the process of economic development. That is, when the proportion of primary industry is less than 10%, and the proportion of secondary industry is less than that of tertiary industry, and the city is in the post-industrialization development stage. The city for which the proportion of primary industry is less than 10% and the proportion of secondary industry is higher than that of tertiary industry is in the late stage of industrialization. The city for which the proportion of primary industry is less than 20% and the proportion of secondary industry is higher than that of tertiary industry is in the middle stage of industrialization. The city for which the proportion of primary industry is higher than 20% and the proportion of primary industry is less than that of secondary industry is in the early stage of industrialization. These three stages are collectively referred to as the stages of industrialization. The development stage of pre-industrialization is characterized by a higher proportion of primary industry than secondary industry [74]. The cities in the first group were in the industrialization stage (60 cities), which is mainly characterized by secondary development. The cities in the other group were in the post-industrialization development stage (44 cities). In this stage, tertiary industry is the leading industry, and the internal structure of the manufacturing industry also changes from capital-intensive to technology-intensive industry. Based on the above grouping of cities, we further explored the impact of the market-oriented reform of industrial land on urban industrial pollution for cities in different stages of industrialization. We included two groups of sample data in the model for estimation, and after the F test and Hausman test, we adopted the fixed effect model to estimate the two groups of sample data. The estimated results are shown in Table 3. The *R*^2^ and F values of the two models were both significant, and the estimation results were good. The estimated results of grouped sample variables were analyzed as follows.

Let us compare the estimated results of control variables in the two groups and the full sample first. In terms of the coefficient of per capita GDP (pgdp), the impact of per capita GDP on urban industrial pollution of cities in the industrialization stage was not significant. For cities in the post-industrialization stage, the impact of per capita GDP on urban industrial pollution was consistent with the full sample model, showing an obvious changing trend of increase first, then decrease, and then increase again. As far as foreign direct investment (fdi) is concerned, with the increase in foreign direct investment, urban industrial pollution may intensify in cities in the industrialization stage. This may have occurred because these cities tend to lower environmental control standards to attract more foreign capital investment to promote economic development, which also leads to the entry of more pollution-intensive and resource-consuming industries, thus resulting in the deterioration of urban environmental quality. The regression coefficients of energy density (ene) of the two groups of sample cities were consistent with those of the full sample, which were significantly positive. The industrial structure (ind) of the two groups of sample cities had a significantly positive influence on urban industrial pollution, and this was consistent with the full sample. However, it was not significant in sample cities at the stage of industrialization. This may have occurred because the industrial scale of such cities is relatively large, and the scale economy effect was obvious. With the expansion of the industrial scale, the increase in urban industrial pollution in these cities was relatively small.

As for the main explanatory variables reflecting the market-oriented reform of industrial land conveyance, the impact of industrial land price (ilp) on urban industrial pollution was basically consistent with the full sample. That is, no matter whether the city was in the stage of industrialization or post-industrialization, along with the increase in industrial land price, urban industrial pollution tended to increase first, and then decrease, and this was statistically significant. However, there was a significant difference in the inflection point of this inverted U-shaped change rule in cities of different industrialization stages. The inflection point of cities in the industrialization stage appeared in the land price of 126 yuan/m^2^ (1.26 million yuan/ha), while it was 1134 yuan/m^2^ (11.34 million yuan/ha) for cities in the post-industrialization stage. This may be due to the fact that the industrial land price is generally lower for cities in the industrialization stage, which is the result of investment promotion of these cities. Our data showed that from 2003 to 2016, the average industrial land price in cities of industrialization stage was 376 yuan/m^2^, much lower than the 637 yuan/m^2^ in cities of post-industrialization stage.

The influence of dummy variables reflecting the market-oriented reform of industrial land conveyance (ilm) shows significant differences in cities of different industrialization stages. The influence of the dummy variable on urban industrial pollution was significantly positive for cities in the industrialization stage. To be exact, industrial pollution has risen by 15% since the market-oriented reform of industrial land. This may be because the industrial structure of these cities was still dominated by traditional secondary industry, and large-scale heavy industries producing high levels of pollution still had a comparative advantage in these cities. Therefore, even if the industrial land conveyance method is more open and transparent, there is no other industry that can challenge the development of these industries for the time being. For cities in the post-industrialization stage, the regression coefficient of the dummy variable was consistent with the full sample, which was significantly negative. Specifically, industrial pollution has decreased by about 38% since the market-oriented reform of industrial land. It shows that the overall industrial structure of these cities was relatively high, and the technology and knowledge-intensive industries with a higher productivity have gradually replaced the labor and capital-intensive industries with lower productivity. Therefore, the openness and transparency of industrial land conveyance will effectively inhibit the development of low-end industries with low productivity, and this plays a significant role in reducing urban industrial pollution.

## 5. Conclusions and Policy Implication

### 5.1. Conclusions

Industrial pollution control is a difficult problem in China’s current economic transformation, and the Chinese government has implemented many measures to deal with it. However, little research has focused on the relationship between land policy and industrial pollution. Based on the theoretical discussion of the mechanism influencing the market-oriented reform of industrial land on urban industrial pollution, we constructed an analytical framework by linking land policy with industrial pollution. Then, we constructed an econometric model reflecting the influence of the market-oriented reform of industrial land on urban industrial pollution and chose the statistical data of 104 large- and medium-sized cities in mainland China from 2003 to 2016. The results indicate that the impact of the market-oriented reform of industrial land conveyance on urban industrial pollution is gradually emerging. With the marketization of the industrial land conveyance price, urban industrial pollution is presenting an inverted U-shaped change trend of increase first, and then decrease. The inflection point of the above inverted U-shaped change rule appears in the industrial land price of about 274 yuan/m^2^ (2.74 million yuan/ha). The price mechanism has played a significant role in regulating the pollution emissions of industrial enterprises. The openness of industrial land conveyance can also significantly reduce urban industrial pollution. For cities in different development stages of industrialization, there is no difference in the impact of industrial land conveyance price on urban industrial pollution. As China’s industrial land prices are generally low, industrial pollution will increase first and then decrease with the increase in industrial land prices. However, the openness of industrial land conveyance promotes and inhibits the urban industrial pollution in the stages of industrialization and post-industrialization, respectively. The economic development level, foreign direct investment, energy intensity, industrial structure, and other social and economic development factors have also had a significant impact on China’s urban industrial pollution.

### 5.2. Policy Implication

The above conclusions have important guiding significance for promoting the market-oriented reform of industrial land conveyance and reducing urban industrial pollution. Therefore, we provide the following two policy recommendations. Firstly, it is necessary to further improve the market-oriented allocation mechanism of industrial land. Our research conclusions show that with the promotion of the market-oriented reform of industrial land conveyance, the industrial land price gradually returns to the market level and the industrial land conveyance is more open, which can screen urban industrial enterprises and ultimately effectively reduce urban industrial pollution. Therefore, for Chinese governments at all levels, it is still necessary to continuously promote the market-oriented reform of industrial land, take full advantage of the market mechanism in optimizing the allocation of industrial land, and regulate the land use behavior of enterprises to effectively curb industrial pollution. Secondly, it is also necessary for the government to pay attention to the regulation of the land market. Through empirical research, we found that in cities in the industrialization stage, large-scale traditional industries producing high levels of pollution still occupy the dominant position, so the simple market-oriented reform of industrial land will fail to curb industrial pollution. In this regard, we suggest that, in the process of promoting the market-oriented reform of industrial land conveyance, the government could also set some control measures for the purpose of public interest and environmental protection to help small- and medium-sized enterprises with high technology levels and low levels of pollution to gain advantages in obtaining industrial land.

## Figures and Tables

**Table 1:** Descriptive statistics of the variables used in the model.

Variable	Unit	Number of Observations	Mean	Std. Deviation	Minimum	Maximum
*poll*	t/10^8^ yuan	1444	61.54	85.86	0.16	723.97
*ilp*	yuan/m^2^	1456	486.86	318.17	68.00	3742.00
*ilm*	-	1456	0.71	0.45	0	1
*pgdp*	yuan	1456	42,827.86	29,132.67	3393	167,411
*fdi*	10^4^ dollars	1456	140,953.30	252,097.68	0.00	3,082,563.00
*ene*	kW·h/10^4^ yuan	1419	670.23	735.47	24.99	11,208.10
*ind*	%	1456	49.23	9.57	18.57	85.92

Note: poll: the emission intensity of industrial SO_2_; ilp: the urban industrial land price; ilm: the dummy variable of the reform of industrial land conveyance mode; pgdp: per capita GDP; fdi: foreign direct investment; ene: the consumption intensity of electricity; ind: the industrial structure (the proportion of the secondary industry output value to gross regional product).

**Table 2:** Estimation results of full sample model.

Variables	Dependent Variable: In *Poll*	
	Fixed Effect	Random Effect
constant	59.509 ** (24.633)	55.009 ** (24.702)
lnilp	2.738 *** (0.529)	2.575 *** (0.519)
${\left(\ln ilp\right)}^{2}$	−0.244*** (0.044)	−0.227 *** (0.043)
ilm	−0.011 (0.045)	−0.070 (0.043)
lnpgdp	−16.985 ** (7.196)	−15.984 ** (7.215)
${\left(\ln pgdp\right)}^{2}$	1.594 ** (0.704)	1.508 ** (0.705)
${\left(\ln pgdp\right)}^{3}$	−0.054 ** (0.023)	−0.051 ** (0.023)
lnfdi	−0.063 *** (0.019)	−0.059 *** (0.018)
lnene	0.235 *** (0.420)	0.323 *** (0.039)
ind	0.008 *** (0.003)	0.010 *** (0.003)
$R^{2}$	0.655	0.675
F/Wald chi2	F = 630.19,	Wald chi2 (10) = 5638.89
N	1403	1403
Hausman test	Prob > chi2 = 0.0000	

Note: Standard errors are in parentheses. ***, **, * indicate significance at 1%, 5%, and 10% respectively.

**Table 3:** Estimation results of grouped sample model.

Variables	Dependent Variable: In *Poll*	
	Industrialization Stage (Fixed Effect)	Post-Industrialization Stage (Fixed Effect)
constant	−4.925 (30.351)	171.060 *** (54.323)
lnilp	1.876 ** (0.844)	5.725 *** (0.913)
${\left(\ln ilp\right)}^{2}$	−0.194 *** (0.072)	−0.407 *** (0.069)
ilm	0.144 ** (0.057)	−0.324 *** (0.067)
lnpgdp	3.262 (8.942)	−54.054 *** (15.502)
${\left(\ln pgdp\right)}^{2}$	−0.396 (0.885)	5.226 *** (1.480)
${\left(\ln pgdp\right)}^{3}$	0.010 (0.029)	−0.171 *** (0.047)
lnfdi	0.004 (0.023)	−0.173 *** (0.030)
lnene	0.152 *** (0.052)	0.318 *** (0.063)
ind	0.002 (0.004)	0.041 *** (0.005)
$R^{2}$	0.674	0.628
F	F = 585.24,	F = 260.32,
N	802	601

Note: Standard errors are in parentheses. ***, **, * indicate significance at 1%, 5%, and 10% respectively.
